# Neuronal overexpression of hTDP-43 in *Caenorhabditis elegans* impairs different neuronally controlled behaviors and decreases fecundity

**DOI:** 10.17912/micropub.biology.000769

**Published:** 2023-04-19

**Authors:** Mandy Koopman, Lale Güngördü, Renée I. Seinstra, Ellen A.A. Nollen

**Affiliations:** 1 European Research Institute for the Biology of Ageing, University of Groningen, University Medical Centre Groningen, Laboratory of Molecular Neurobiology of Ageing, The Netherlands; 2 European Research Institute for the Biology of Ageing, University of Groningen, University Medical Centre Groningen, The Netherlands

## Abstract

Cytoplasmic inclusions consisting of transactive response DNA-binding protein 43 (TDP-43) are a key hallmark of TDP-43 proteinopathies like amyotrophic lateral sclerosis (ALS). *Caenorhabditis elegans* is considered a useful model for studying the molecular mechanisms underlying TDP-43 toxicity *in vivo* . Here, we assessed different neuronal systems through established behavioral assays and extended the phenotypic characterisation of a *C. elegans* model expressing wildtype human *TDP-43* ( *hTDP-43* ) pan-neuronally. Our data show that neuronal expression of hTDP-43 in *C. elegans* disrupts chemotaxis and decreases fecundity. The basal slowing response, on the other hand, appears to be preserved in the presence of hTDP-43.

**Figure 1. Expression of hTDP-43 in C. elegans alters several sensorimotor phenotypes:**
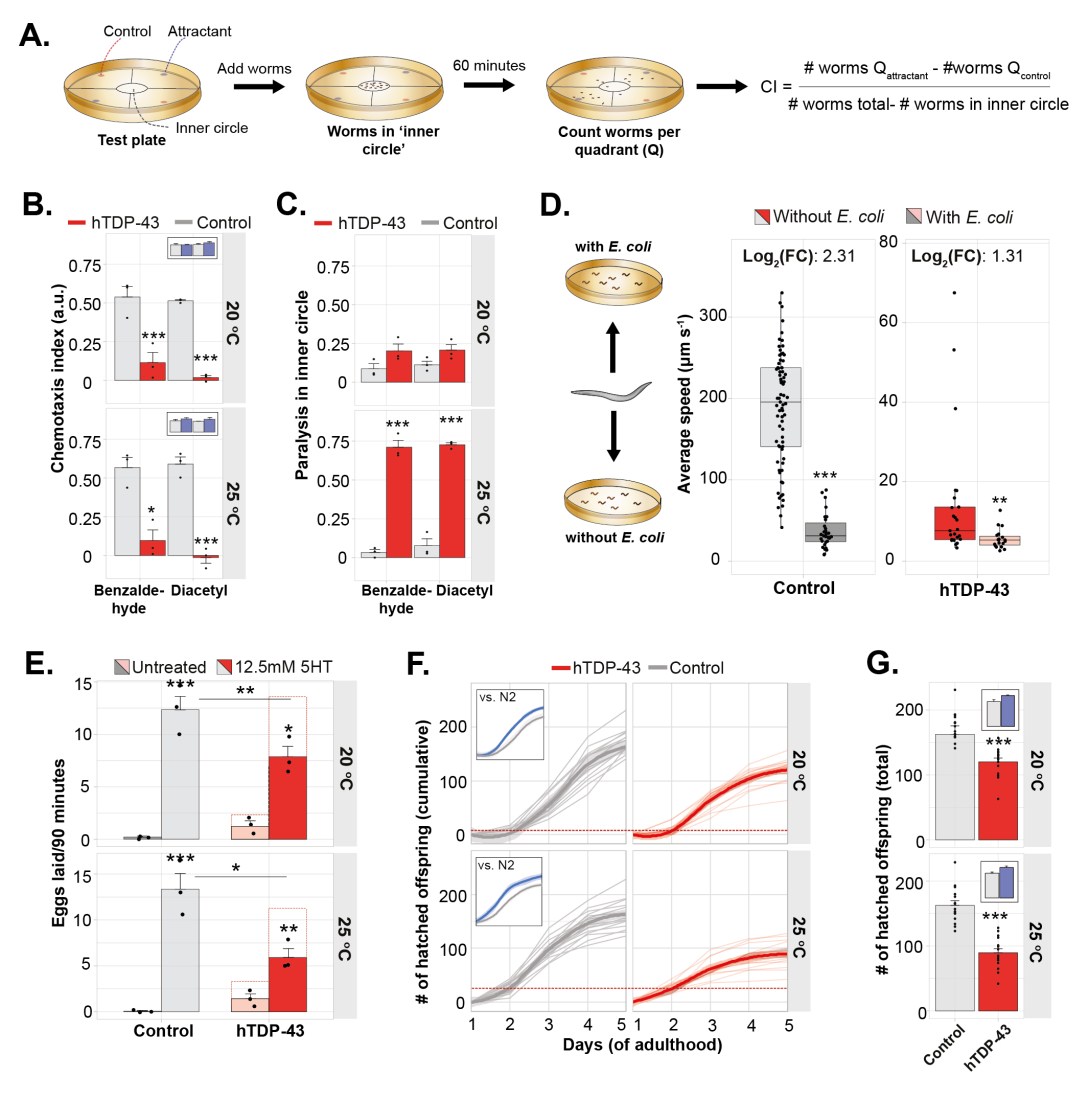
**A)** Schematic showing the experimental outline for the chemotaxis assay and the formula used to calculate the chemotaxis index (CI). Genotype of hTDP-43 worms: *snb-1p::hTDP-43/3’long UTR + mtl-2p::GFP* , genotype of control worms: *mtl-2p::GFP.* **B)** Chemotaxis assay assessing the chemotactic index towards 0.5% benzaldehyde or diacetyl. Small insert shows comparison between N2 and control worms. *n = 3,* two-way ANOVA at 20 °C and 25 °C (treatment: n.s., genotype: p<0.001, interaction: n.s.) with post-hoc Sidak’s multiple comparison test. **C)** The paralysis index of worms that are unable to leave the start-circle is shown. *n = 3* two-way ANOVA at 20 °C (treatment: n.s., genotype: p = 0.016, interaction: n.s.) with post-hoc Sidak’s multiple comparison test, benzaldehyde: n.s. diacetyl: n.s. Two-way ANOVA at 25 °C (treatment: n.s., genotype: p <0.001, interaction: n.s.) with post-hoc Sidak’s multiple comparison test, benzaldehyde. **D)** The basal slowing response of well-fed animals. The average crawling speed (μm s ^-1^ ) of hTDP-43 and control worms, *n* = 19-73, Mann-Whitney U test hTDP-43: p = 0.002, Control: p<0.001. One representative experiment of a triplicate is shown. **E)** 5-HT mediated egg-laying behavior in liquid. *n = 3,* two-way ANOVA at 20 °C (treatment: p<0.001 genotype: n.s. interaction: p = 0.012) and 25 °C (treatment: p<0.001, genotype: p = 0.017, interaction: p = 0.003.) with post-hoc Tukey multiple comparison test. Dotted line bars represent the number of eggs laid after correction of the data by the results shown in G. **F)** Number of hatched offspring produced by hTDP-43 and control worms. The accumulation of hatched offspring per worm is represented in the accumulative line-graph and **G** ) The total number of hatched offspring in the period from D1-D5 of adulthood is represented as bar-chart. Small inserts show comparison between N2 and control worms. *n = 15,* two-tailed unpaired Student’s t-test for both 20 °C and 25 °C: p<0.001. One representative experiment of a triplicate is shown. Worms were grown from L1 at 20 °C and subsequently transferred at D1 to 20 °C or 25 °C. Error bars represent the S.E.M. and transparent areas in line-graphs represent 95% confidence interval. *: p ≤ 0.05, **: p ≤ 0.01, ***: p ≤ 0.001.

## Description

Given the clear proteotoxicity of TDP-43 in the neuromuscular system (Koopman et al., 2023b), we subsequently investigated other characteristic neuronal controlled behaviors. Two pairs of amphid sensory neurons, AWC and AWA, and multiple interneurons are required for chemotaxis to volatile odors like benzaldehyde and diacetyl (Bargmann et al., 1993; Hobert et al., 2003) . The chemotaxis index (CI) is a measure for the fraction of worms that can arrive at the location of the attractants (Matsuura et al., 2005) ( **Figure 3A** ). To investigate chemotaxis behavior, we assayed the CI towards both benzaldehyde and diacetyl in hTDP-43 worms ( **Figure 3B** ). At 20 °C and 25 °C, hTDP-43 worms exhibit a significant reduction of the CI towards both attractants compared with the control strain. At 25 °C, however, the CI was highly confounded by a high paralysis rate which is reflected by a large fraction of hTDP-43 worms that did not enter a test quadrant at all ( **Figure 3C** ). Nevertheless, our results generally indicate that the circuit required for chemotaxis is affected by neuronal expression of hTDP-43.

Next, we assayed the basal slowing response, which relies on dopaminergic neurons that transduce a mechanosensory stimulus through interneurons and motoneurons to effect slowing of the locomotory rate ( **Figure 3D** ). The ‘basal slowing response’ refers to the observation that well-fed animals transferred to plates containing bacteria normally move more slowly than those transferred to assay plates without bacteria (Sawin et al., 2000) . Since the speed of hTDP-43 grown at 25 °C is virtually zero ( **Figure 2C** ), we only assayed worms raised at 20 °C. We measured the average speed of hTDP-43 and control worms on plates with and without *E. coli* and found that both groups significantly reduced their speed on bacteria ( **Figure 3D** ). The slowing effect was, however, less pronounced in hTDP-43 worms as evidenced by the log2 fold-change in speed ( *Cohen’s d _control_ =* 2.52, *d _TDP-43_ =* 0.70) ( **Figure 3D** ). The reduced food-slowing effect may represent a true biological finding or, due to a lower empty-plate based speed, could be a mere consequence of the reduced response-window for food-slowing of hTDP-43 worms ( **Figure 3D** ). The latter is not unlikely given the comparable speed of hTDP-43 and controls worms on food ( **Figure 3D** ). Thus, our results show that hTDP-43 worms can functionally slow on food, though with a smaller drop in locomotion speed than control worms.

Normally, when food-deprived worms are transferred to bacteria they display a dramatically enhanced slowing response. This experience-dependent response is mediated by serotoninergic neurotransmission (Sawin et al., 2000) . Since hTDP-43 worms have an average speed close to 0 µm/s on food, it is impossible to measure the ‘enhanced slowing response’. Consequently, we opted for an alternative way to investigate serotonergic neurons and focused on serotonin-dependent egg-laying. Taking advantage of the ability of *C. elegans* to take up exogenously applied serotonin (5-HT) we conducted a 5-HT-induced egg-laying paradigm to determine whether serotonergic neurotransmission is affected by hTDP-43 expression (Trent et al., 1983; Desai et al., 1988; Schafer et al., 1996) . Under normal conditions, control animals lay few eggs when transferred to M9-buffer but addition of 5-HT to the media increases the rate of egg laying (Carnell et al., 2005) ( **Figure 3E** ). 5-HT had a reduced effect on the egg-laying rate of hTDP-43 worms ( **Figure 3E** ). To assess whether the altered response to 5-HT could be partially due to lower fecundity, we followed hTDP-43 worms and their controls over time and counted the number of hatched offspring. We found that hTDP-43 worms produce less offspring than control animals and that this effect was enhanced at 25 °C ( **Figure 3F, G** ). When the results of the egg-laying paradigm are corrected for the observed decrease in fecundity (i.e. hatched offspring) the differences between control and hTDP-43 worms are no longer significant ( **Figure 3E, red dotted lines** versus uncorrected control). It should, however, be noted that there is a clear difference between the 'total eggs laid' and 'hatched offspring', as some eggs may never hatch. Therefore, the corrected 5-HT egg-laying should be interpreted with caution, as differences in the ratio eggs laid:hatched offspring were not specifically quantified in hTDP-43 versus control worms.

Taken together, our results show that hTDP-43 disrupts chemotaxis, decreases fecundity and potentially modifies the rate of egg-laying. The dopamine-dependent basal slowing was also observed, although the reduction was not as large when compared to control animals. Thus, proteotoxicity of hTDP-43 seems not to be limited to the neuromuscular system.

## Methods

**Strains and maintenance**

Standard conditions were used for *C. elegans* propagation at 20 °C (Brenner, 1974) Animals were age-synchronized by hypochlorite bleaching and subsequently allowed to hatch overnight in M9 buffer at 20 °C. For experiments age synchronized L1s were cultured for 72h at either 20 °C or 25 °C on NGM plates seeded with OP50 before being tested, unless stated differently.

**Hatched offspring**

Synchronized worms were cultured on standard NGM plates seeded with OP50 at 20 °C. Adulthood Day 1 worms were serially transferred (at either 20 °C or 25 °C) to fresh 6-cm NGM plates every 24 h until being sterile. 2-3 d after the eggs were laid, the hatched progeny was counted. In each experiment, 15 adult worms per condition were assessed.

**Serotonin-induced egg laying**

Egg-laying experiments were performed as previously described by Carnell et al., 2005. In short, age-matched adults (for the 20 °C condition: adult day 2, for 25 °C: adult day 1) were placed into 50 μL of M9 buffer with or without 12.5 mM 5-HT (Sigma-Aldrich). The number of eggs laid in each well was counted after 90 minutes. Each experiment represents the average number of eggs laid per 90 minutes of 15 individual worms.

**Basal slowing response**

Food-slowing was performed as previously described by Sawin et al., 2000. In short, well-fed animals were placed at either an empty assay plate (‘control’ condition) or an assay plate with a ring-shaped bacterial lawn (‘food-slowing’ condition). After 5 minutes of acclimatization worms were recorded for 1 minute (1200 frames, 20fps) with the WF-NTP (Koopman et al., 2020) .

**Chemotaxis**

The chemotaxis assay protocol was adapted from Margie et al., 2013. Briefly, the assay was performed in 6 cm Petri dishes containing 8 ml of NGM. These plates were divided into four equal quadrants and an inner circle of approximately 1 cm in diagonal. In two opposing diagonal quadrants a test solution of either 0.5% benzaldehyde (SAFC, W212717) or 0.5% diacetyl (SAFC, W237035) + 0.25M sodium azide (in ethanol) was pipetted. Ethanol mixed with sodium azide was used as control in the other quadrants. Then, worms were transferred in the middle circle and allowed to roam. After 60 minutes pictures were taken and the number of worms per quadrant were counted. Animals were excluded from the analysis if they failed to clear the inner circle or were sitting on the marked lines. The chemotaxis index (CI) was calculated for each strain and experiment using the following formula: CI = (number of animals in both test quadrants – number of animals in both control quadrants)/(total number of scored animals). 150-250 worms per assay were used.

## Reagents

**Table 1:** Strains used

**Table:** 

**Strain**	**Description**	**Genotype**	**Remark**
N2	Bristol wild isolate	Wildtype	
OW1601	CL6049 6x backcrossed with N2 . Named: hTDP-43 worms.	*dvIs62* [ *snb-1p::hTDP-43/3’long UTR + mtl-2p::GFP* ]X	Provided by Chris Link
OW1603	CL2122 6x backcrossed with N2 . Named: control worms.	*dvIs15* [( *pPD30.38) unc-54 (vector) + (pCL26)mtl-2p::GFP* ]	Provided by Chris Link
